# UPSIDES Peer-Begleitung – Gemeinsam Krisen bewältigen

**DOI:** 10.1055/a-1395-1505

**Published:** 2021-04-08

**Authors:** Maria Haun, Rebecca Nixdorf, Dr. Imke Heuer, Maria Wagner, Stefan Bilmayer, Dr. Candelaria Mahlke, Prof. Dr. Bernd Puschner

## Abstract

**Im Rahmen von Peer-Begleitung (engl. peer support) erhält eine Person mit einer psychischen Erkrankung Unterstützung von einer Person, die selbst Erfahrung mit Krisen und/oder psychiatrischen Diagnosen gemacht und damit einen guten Umgang gefunden hat. „Peer-Begleiter/innen helfen als Betroffene Betroffenen“, erklärt Stefan Bilmayer, UPSIDES Peer-Begleiter am deutschen Standort Ulm/Günzburg.**

UPSIDES („Using Peer Support In Developing Empowering Mental Health Services“) ist ein Zusammenschluss von acht Kooperationspartnern in Afrika, Asien und Europa. In UPSIDES wird erstmals auf globaler Ebene eine gemeinsame Peer Support Intervention implementiert und evaluiert 1 . Gefördert wird das Projekt mit einer Laufzeit von fünf Jahren durch die Europäischen Union (EU) und die Global Alliance of Chronic Diseases (GACD).

**Abb. 1:**
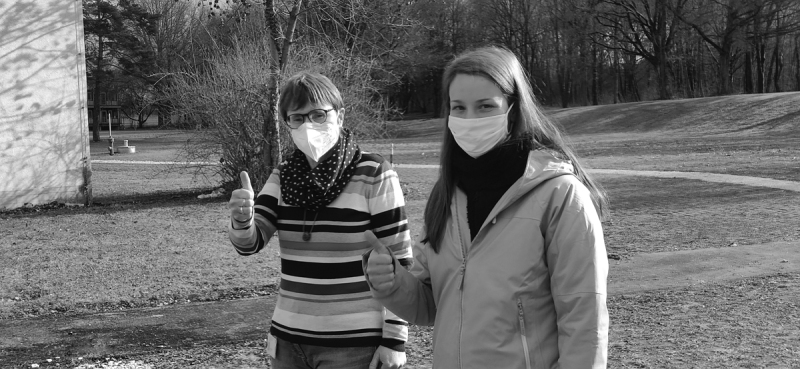
Zwei UPSIDES Peer-Begleiterinnnen am Standort Ulm/Günzburg.

Seit Januar 2020 wird in Tansania, Uganda, Indien und Israel, sowie auch an den beiden deutschen Standorten in Günzburg/Ulm und Hamburg UPSIDES Peer-Begleitung angeboten und in einer randomisierten, kontrollierten Studie evaluiert 2 . Die UPSIDES Peer-Begleitung beinhaltet z. B. Alltagshilfe, Begleitung zu Terminen, Einzelgespräche oder Unterstützung bei der Freizeitgestaltung. In ihrer Arbeit unterstützt werden die Peer-Begleiter/innen durch Supervision und Intervision, sowie durch Teamsitzungen, die aufgrund von Corona zurzeit auch digital stattfinden.

Im Rahmen von UPSIDES wurde ein mehrwöchiges Training für Peer-Begleiter/innen entwickelt. Ziel des Trainings ist es, die Teilnehmer/innen zu befähigen und zu bestärken, auf ihre persönliche Lebenserfahrung zurückzugreifen, um andere Personen in Krisen zu unterstützen. Die UPSIDES Peer-Begleiter/innen am Standort Hamburg haben dazu eine gemeinsame Definition entwickelt: „Unsere Kompetenzen beruhen auf unserer Krisenerfahrung und deren Bewältigung. Wir leiten nicht an, wir begleiten auf Augenhöhe (…) Wir unterstützen Menschen beim Legen der Grundsteine für ein stabiles, hoffnungsvolles und erfülltes Leben“.

Weitere Informationen über UPSIDES finden Sie unter www.upsides.org .
